# Spinal *Leclercia adecarboxylata* infection in an immunocompetent patient: case report

**DOI:** 10.1128/asmcr.00092-24

**Published:** 2025-06-30

**Authors:** Mohannad M. Mallat, Abdullah Alatar, Duaa Alhumoudi, Ali Alhijji, Abdulaziz Alsubaie, Ali Somily, Sherif Elwatidy

**Affiliations:** 1 College of Medicine, King Saud University 191082, Riyadh, Saudi Arabia; 2 Neurosurgery Division, Department of Surgery, King Saud University 37850, Riyadh, Saudi Arabia; 3 Infectious Diseases Division, Department of Medicine, King Saud University 37850, Riyadh, Saudi Arabia; 4 Department of Pathology, College of Medicine, King Saud University 191082, Riyadh, Saudi Arabia; Vanderbilt University Medical Center, Nashville, Tennessee, USA

**Keywords:** *Leclercia adecarboxylata*, spinal infection, immunocompetent patient, soft tissue infection

## Abstract

**Background:**

*Leclercia adecarboxylata* (Family: Enterobacteriaceae) is a gram-negative bacillus that is found in diverse environments but has rarely been isolated from the human microbiota. Notably, no patient with a spinal disc infection caused by *L. adecarboxylata* has been documented to date.

**Case Summary:**

A 60-year-old male presented with chronic lower back pain and decreased extensor hallucis longus muscle power upon examination. Magnetic resonance imaging of the spine revealed canal stenosis at L3-4 and L4-5, along with migrating L4-5 disc material compressing the left lateral recess and foramina. The patient underwent L4-5 decompression and fixation surgery. Culturing of the excised disc material revealed the presence of lactose-fermenting oxidase-negative *L. adecarboxylata*. This isolate was found to be resistant to ampicillin; first-, second-, and third-generation cephalosporins; and trimethoprim–sulfamethoxazole. The patient was started on ciprofloxacin 500 mg twice daily for 6 weeks and initially showed favorable improvement. However, he was subsequently lost to follow-up, preventing further long-term evaluation of his clinical condition.

**Conclusion:**

A literature review revealed documentation of 227 cases of *L. adecarboxylata* infections worldwide, which were reported without a clear pattern but with the highest incidences in Asia. The most common manifestations included bacteremia (22% of patients), soft tissue infections (12.3%), and peritonitis. Notably, 86% of patients recovered, whereas 7% died. This case report and literature review emphasize the importance of accurate identification and treatment, as well as the need for further research to address knowledge gaps on the behavior and management of this pathogen.

## INTRODUCTION

*Leclercia adecarboxylata*, a gram-negative bacillus in the Enterobacterales family, was initially described by H. Leclerc as *Escherichia adecarboxylata* in 1962 but subsequently reclassified to its current name in 1986 (1). Despite its rarity, this bacterium has been historically isolated from diverse environmental reservoirs, food, and animal guts (2). Furthermore, it was found to be a constituent of the human gastrointestinal microbiota (3, 4).

The first-known case of a patient infected with *L. adecarboxylata* was reported in the United States in 1991 by Otani and Bruckner (5), and subsequent reports of other cases have been emerging globally, albeit without a discernible pattern. Furthermore, the disease reportedly manifested as bacteremia, pneumonia, infective endocarditis, peritonitis, septic arthritis, and soft tissue infections (2). In Saudi Arabia, only two *L. adecarboxylata*-infected patients have been reported, one presenting with catheter-related bacteremia and the other with pneumonia (6, 7). Herein, we report a 60-year-old immunocompetent patient with *L. adecarboxylata* infection of the spine and present a literature review on reported cases of infection by this bacterium. Notably, our extensive literature search did not uncover any reports of *L. adecarboxylata* infection of the spinal disc, making this case report noteworthy.

## CASE PRESENTATION

A 60-year-old male, working as a general practitioner, was referred to our neurosurgical clinic at King Saud University Medical City (Riyadh, Saudi Arabia) with an 8-year history of chronic lower back pain radiating to the posterior aspect of the thighs bilaterally. His pain, which had increased in severity over the past 9 months, was aggravated by walking and standing and relieved by sitting and bending forward. Neurogenic claudication was noted at 5–10 m. He also complained of numbness, mainly in the soles of both feet. There was no associated weakness, sphincter dysfunction, or upper motor signs. Any history of trauma was denied. His past medical history was remarkable for hepatitis C that was treated 10 years ago, with no history of intravenous drug abuse. The sequela of the patient’s hepatitis C infection was not fully investigated in our institution. His past surgical history was significant for an appendectomy and a cholecystectomy, which were performed more than 10 years prior. On physical examination, his vitals were stable, and his BMI was 28 kg/m^2^. Neurological assessment revealed a power of 5/5 throughout the limbs, except for decreased power in the extensor hallucis longus muscle (3/5 on the left side and 4/5 on the right). No sensory deficit was found on examination, and both the patellar and Achilles reflexes were +1. The straight leg raise and clonus tests were negative.

**Fig 1:**
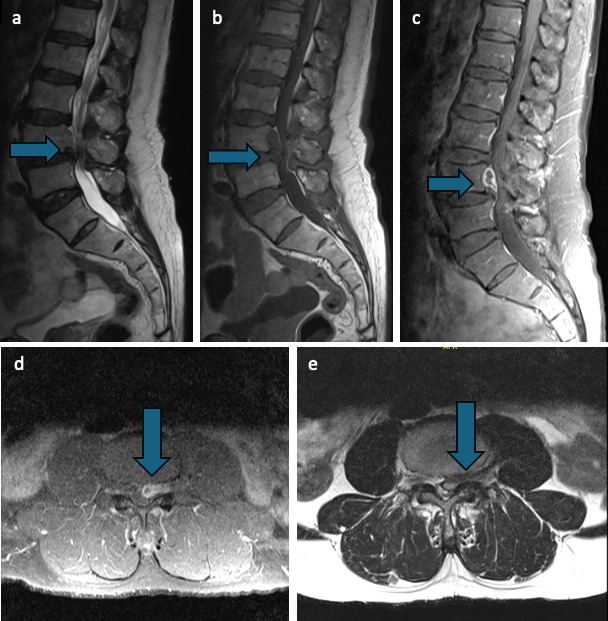
Preoperative magnetic resonance images of sagittal T2 (a), sagittal T1 (b), and sagittal T1 with contrast sequences (c) showing canal stenosis at the level L3-4 and L4-5 vertebrae, with migrating L4-5 disc material behind the L4 vertebral body compressing the left lateral recess and with peripheral contrast enhancement, and axial T1 with contrast (d) and axial T2 sequences (e) showing significant canal stenosis with left L4 foraminal stenosis.

His laboratory results revealed anemia 125 gm/L (130–180 gm/L) but normal white blood cell (WBC) and C-reactive protein (CRP). The patient underwent electromyography and nerve conduction study, both of which showed evidence of subacute to chronic L5-S1 radiculopathy on the left side. Contrast magnetic resonance imaging of the spine showed canal stenosis at the L3-4 and L4-5 vertebrae, with migrating L4-5 disc material behind the L4 vertebral body compressing the left lateral recess and L4 foramina and with peripheral contrast enhancement (Fig. 1). Computed tomography imaging revealed disc degeneration (more pronounced in the L4-5 level), resulting in severe canal and foraminal stenosis.

The patient was admitted and scheduled for L4-5 decompression and fixation surgery. Intraoperatively, laminectomy and discectomy were performed with pedicle screws and rod fixation at L4-5. Because the excised disc material was abnormally yellowish in color and consistency, it was sent for microscopic and histopathological examination. The patient was discharged on day 4 after surgery in good clinical condition, with satisfactory postoperative X-ray results. The excised disc material was inoculated onto 5% sheep blood agar, MacConkey agar plates, and Todd Hewitt broth and incubated at 36 ± 1°C. Two days later, lactose-fermenting oxidase-negative gram-negative bacilli had grown on the solid and liquid media. Bacterial strain identification and antimicrobial susceptibility tests were performed using MicroScan WalkAway Plus (Becton Dickinson, Heidelberg, Germany) according to the manufacturer’s instructions. The antimicrobial susceptibility test results were interpreted according to breakpoints defined by the Clinical and Laboratory Standards Institute (CLSI) (8). The bacterial cells were identified as *L. adecarboxylata* with 100% probability. The isolate was susceptible to aminoglycosides, piperacillin–tazobactam, carbapenems, cefepime, ciprofloxacin, and tigecycline but resistant to ampicillin, first-, second-, and third-generation cephalosporins and trimethoprim–sulfamethoxazole (Table 1). Subsequently, the patient was started on ciprofloxacin 500 mg twice daily for 6 weeks. Upon initial follow-up in the clinic after 2 weeks, the condition of the patient was favorable. However, because follow-up was lost thereafter, further clinical, laboratory, and radiological assessments to determine his full antibiotic response could not be performed.

**TABLE 1:** Antibiotic susceptibility and resistance of *L. adecarboxylata^a^*

Drug	Result	CLSI MIC breakpoints for Enterobacterales (μg/mL)		
		S	I	R
Ampicillin	R	≤8	16	≥32
Cefepime	S	≤2	–^*b*^	≥16
Cefotaxime	R	≤1	2	≥4
Ciprofloxacin	S	≤0.25	0.5	≥1
Ertapenem	S	≤0.5	1	≥2
Gentamicin	S	≤2	4	≥8
Imipenem	S	≤1	2	≥4
Meropenem	S	≤1	2	≥4
Trimethoprim–sulfamethoxazole	R	≤2/38	–	≥4/76

^*a*^ *L. adecarboxylata* showed resistance to ampicillin, cefotaxime, and trimethoprim–sulfamethoxazole. Minimum inhibitory concentration (MIC) results were interpreted according to breakpoints defined by the CLSI Performance Standards for Antimicrobial Susceptibility Testing, 35th ed., CLSI M100-ED35:2025. S, susceptibility; R, resistance.

^*b*^ –, no breakpoint measurement.

## DISCUSSION

*L. adecarboxylata* has previously been isolated from various environments, such as water, soil, the surface of chicken eggshells, and the oral cavities of sharks (2). Clinically, the bacterium has been isolated from a diverse array of human biological specimens, namely, blood, urine, wound swabs, deep tissue, gallbladder, peritoneal fluid, cerebrospinal fluid, synovial fluid, respiratory tract, bone, and abscess aspirate (2).

Colonies of this facultative anaerobic bacterium typically appear gray and smooth in texture on blood agar, with some strains producing a yellow to light-yellow pigment, which can be a distinguishing feature. On MacConkey agar, the organism is typically identified as a lactose fermenter, producing pink colonies due to lactose fermentation and subsequent acid production (9, 10).

Although the clinical characteristics of *L. adecarboxylata* remain unclear, its low pathogenicity and high antibiotic susceptibility suggest that it is primarily non-lethal (6). Several hypotheses regarding its possible routes of infection have been proposed: for example, bacterial translocation from the genitourinary tract, direct host access through catheters and wounds, and bacterial translocation across mucosal barriers of the gastrointestinal tract (2, 3, 11). *L. adecarboxylata* tends to be part of a polymicrobial infection in immunocompetent patients (12, 13). However, it can present as a monomicrobial infection in immunocompromised patients, particularly those with predisposing factors such as peritoneal dialysis, central lines, wounds, and other immunosuppression factors (2, 12, 14, 15). Interestingly, although our patient was immunocompetent, he presented with a monomicrobial intervertebral disc infection.

*L. adecarboxylata* is likely underreported in the literature (2), particularly in countries with minimally equipped laboratories that do not have access to automated identification systems, such as matrix-assisted laser desorption/ionization time-of-flight mass spectrometry and 16S rRNA or whole-genome sequencing, and are instead using Analytical Profile Index for Enterobacterales and Vitek 2 software that have yet to be updated to accommodate new bacterial strains (2, 16, 17). *L. adecarboxylata* can potentially be misidentified when using conventional methods because it exhibits very similar yields and properties to those of other species from the same family of Enterobacterales, such as *Escherichia coli* (7, 17).

The breakpoints of antibiotics are specific for each bacterial species, and some laboratories hold off susceptibility testing until the identity of the isolate is confirmed. Given the rarity of *L. adecarboxylata*, the challenge posed in its identification may lead to a delay in susceptibility testing and in devising the optimal antibiotic regimen. Additionally, despite its excellent antibiotic susceptibility profile, strains resistant to antibiotics such as fosfomycin and ampicillin are emerging (18, 19). In our patient, the strain was sensitive to ciprofloxacin, meropenem, imipenem, and gentamicin and resistant to ampicillin, cefotaxime, and trimethoprim–sulfamethoxazole. Our antibiotic of choice was ciprofloxacin 500 mg twice daily for 6 weeks. Unfortunately, the patient was lost to long-term follow-up but was responding well to treatment at his postoperative 2-week assessment.

We conducted a literature review of all reported human cases of *L. adecarboxylata* infection using three databases: PubMed, Scopus, and Google Scholar. English and non-English (translated) articles were retrieved using the search terms “*Leclercia adecarboxylata*,” “*L. adecarboxylata*,” and “*Escherichia adecarboxylata*.” Each publication was separately reviewed by two authors. The most recent search was conducted on 7 March 2024. In total, 96 publications covering 227 patients infected with *L. adecarboxylata* were identified (Table 2).

**TABLE 2:** Individual case reports, outbreaks, and descriptive studies of *L. adecarboxylata* infections from 1991 to 2023^*a*^

Authors	No. of pts	Year	Sex	Age^*b*^	Country	Clinical presentation	Isolation source	Risk factors	Immunity status	Outcome	Polymicrobial infection^*c*^
Otani and Bruckner (5)	1	1991	M	8 m	USA	Bacteremia	Blood	Gastroschisis, intestinal atresia	Unk	Recovery	−
Cai et al. (20)	3	1992	Unk	Unk	China	Diarrhea	Blood, feces, gallbladder	Previous cardiac surgery	Unk	Unk	−
Daza et al. (21)	1	1993	M	45	Spain	Bacteremia	Liver	Hepatic cirrhosis	Compromised	Death	−
Dudkiewicz and Szewczyk (22)	Uk	1997	Unk	Unk	Poland	Endocarditis	Cardiac valve	Unk	Unk	Unk	Unk
Temesgen et al. (15)	5	1997	F	35	USA	Neutropenia	Blood	Leukemia	Compromised	Death	−
			M	54		Pneumonia	Sputum	Esophagitis, degenerative arthritis, Still’s disease	Compromised	Recovery	+
			M	23		Soft tissue infection	Wound	None	Competent	Recovery	+
			M	35		Soft tissue infection	Wound	None	Competent	Recovery	+
			F	43		Soft tissue infection	Wound	None	Competent	Recovery	+
Martínez et al. (23)	1	1998	M	78	Spain	Foot ulcer	Wound	DM	Compromised	Unk	−
Hwang et al. (24)	1	1998	M	60	South Korea	Peritonitis	Peritoneal fluid	PD, ESRD	Compromised	Unk	−
Lee et al. (25)	1	1999	F	69	South Korea	Bacteremia	Blood, catheter tip	Chemotherapy	Compromised	Unk	+
de la Obra et al. (26)	1	1999	F	42	Spain	Bacteremia	Blood	MM	Compromised	Recovery	−
Fattal and Deville (27)	1	2000	M	5	USA	Peritonitis	Peritoneal fluid	PD, ESRD	Compromised	Recovery	−
de Baère et al. (28)	2	2001	F	78	Belgium	Cholecystitis	Gallbladder	*Candida albicans* sepsis, pneumonia, previous ERCP	Compromised	Recovery	+
			F	80		Bacteremia	Blood	*Candida albicans* sepsis, coronary bypass, pneumonia	Compromised	Recovery	+
Greco et al. (29)	3	2001	M	38	Argentina	Bacteremia	Blood	Renal transplant, dialysis	Compromised	Unk	−
			M	77		Soft tissue infection	Wound	Colon tumor	Compromised	Unk	+
			M	75		Soft tissue infection	Wound	None	Unk	Unk	+
Rodríguez et al. (30)	1	2001	M	74	Spain	Peritonitis	Peritoneal fluid	PD	Compromised	Unk	+
Longhurst and West (31)	1	2001	F	11 m	USA	Bacteremia	Blood	ALL	Compromised	Recovery	+
Pérez-Moreno et al. (32)	1	2003	M	60	Spain	Septic arthritis	Synovial fluid	None	Competent	Recovery	−
Mazzariol et al. (33)	1	2003	M	58	Italy	Bacteremia	Blood	AML	Compromised	Recovery	−
Beltrán et al. (34)	1	2004	F	61	Spain	Soft tissue infection	Wound	DM, SVI	Compromised	Recovery	+
Sawamura et al. (35)	1	2005	M	59	Japan	Pyelonephritis	Urine	Bladder cancer	Compromised	Recovery	+
Kim et al. (13)	1	2008	M	71	South Korea	Peritonitis	Peritoneal fluid	HCC and cirrhosis	Compromised	Recovery	−
Jover-Sáenz et al. (36)	1	2008	F	81	Spain	Cholecystitis	Bile	Metabolic syndrome, atrial fibrillation	Compromised	Recovery	−
Hess et al. (12)	1	2008	F	40	USA	Soft tissue infection	Wound	None	Competent	Recovery	−
Dalamaga et al. (37)	1	2008	M	30	Greece	Epididymo-orchitis	Blood, urine	T10 paraplegia	Compromised	Recovery	+
Lee et al. (11)	1	2009	F	48	South Korea	Endocarditis, bacteremia	Blood	Endometrial cancer	Compromised	Recovery	−
Dalamaga et al. (38)	1	2009	M	53	Greece	Soft tissue infection, bacteremia	Blood	Burn	Compromised	Recovery	−
Corti et al. (39)	1	2009	M	37	Italy	Soft tissue infection	Wound	Trauma	Competent	Recovery	+
Fernández-Ruiz et al. (40)	2	2009	M	72	Spain	Catheter-related bacteremia	Blood	Hemodialysis, transplant	Compromised	Recovery	+
			M	81		Catheter-related bacteremia	Blood	DM, hemodialysis	Compromised	Recovery	−
Marco Lattur et al. (41)	1	2010	M	22	France	Bacteremia	Blood	Ewing sarcoma with metastasis, PE	Compromised	Recovery	−
Shah et al. (42)	1	2011	M	8	USA	Soft tissue infection	Wound	ALL	Compromised	Recovery	−
Marina et al. (43)	1	2011	M	58	USA	Catheter-related bacteremia	Blood	ESRD, DM	Compromised	Recovery	−
Tam and Nayak (44)	1	2012	M	81	USA	Soft tissue infection	Wound	DM, hypertension	Compromised	Recovery	+
Shin et al. (45)	1	2012	F	47	South Korea	Bacteremia	Blood	Breast cancer	Compromised	Recovery	−
Myers et al. (46)	1	2012	F	7 d	Canada	Sepsis	Blood	Prematurity	Compromised	Recovery	−
Forrester et al. (47)	1	2012	M	55	USA	Bacteremia, septic shock	Blood	Trauma	Competent	Recovery	+
Michael et al. (48)	1	2013	M	25	USA	Soft tissue infection	Wound	Trauma, foreign body, explosives	Competent	Recovery	−
Nelson et al. (49)	1	2013	F	1 m	USA	Sepsis, gastric perforation	Blood	Prematurity, RDS	Compromised	Death	−
Eiland et al. (50)	1	2013	F	55	USA	Acute respiratory and renal failure	Bronchial wash	Leukopenia	Compromised	Death	−
De Mauri et al. (51)	1	2013	M	81	Italy	Catheter-related bacteremia	Blood	Hemodialysis	Compromised	Recovery	−
Bali et al. (52)	1	2013	M	32	India	Peritonsillar and pharyngeal space abscess + SIRS)	Oral cavity	None	Competent	Recovery	−
Sethi et al. (3)	1	2014	M	5	USA	Sepsis	Blood	Intestinal inertia, central line	Compromised	Death	−
Sanchez Porto et al. (53)	1	2014	F	56	Spain	Soft tissue infection, bacteremia	Blood	Metabolic syndrome	Compromised	Recovery	−
Keren et al. (14)	1	2014	M	46	Israel	Soft tissue infection	Wound	None	Competent	Recovery	+
Kashani et al. (4)	1	2014	F	43	USA	Ulcerative colitis, enteropathic arthritis	Colon	IBD	Compromised	Recovery	−
García-Fulgueiras et al. (54)	1	2014	M	59	Uruguay	Necrotizing lesion, osteomyelitis	Bone	DM, Liver disease	Compromised	Unk	−
Haji et al. (55)	1	2014	M	70	Japan	Septic arthritis, Bacteremia	Blood	None	Competent	Recovery	−
Chao et al. (56)	1	2014	F	48	Taiwan	Peritonitis	Peritoneal fluid	PD	Compromised	Recovery	−
Anuradha (57)	2	2014	F	31	India	Vaginitis	Vaginal swab	None	Competent	Recovery	−
			M	50		Soft tissue infection (gluteal abscess)	Pus discharge	None	Competent	Recovery	−
Prakash et al. (58)	3	2015	M	41	India	Pneumonia	Tracheal aspirate	Head trauma	Competent	Recovery	−
			F	32		Pneumonia	Tracheal aspirate	-	Competent	Recovery	−
			M	32		Pneumonia	Tracheal aspirate	HIV, spinal TB	Compromised	Death	−
Hurley et al. (59)	1	2015	M	2	USA	Soft tissue infection	Wound	None	Competent	Recovery	−
Grantham et al. (60)	1	2015	F	9	USA	Soft tissue infection	Wound	None	Competent	Recovery	+
Allawh and Camp (61)	1	2015	F	26	USA	Soft tissue infection	Wound	None	Competent	Recovery	+
Papacharalampous et al. (62)	1	2015	M	57	Greece	Infective aortic aneurysm	Blood, abscess	COPD	Competent	Recovery	−
Jean et al. (63)	1	2016	M	66	Taiwan	Bacteremia	Blood	Long term use of NSAIDs	Compromised	Recovery	−
Voulalas et al. (64)	1	2016	M	55	Greece	Mycotic aneurysm	Bone	None	Compromised	Recovery	−
Zamora (65)	1	2016	F	34	USA	Soft tissue infection, bacteremia	Blood	ESRD, RHD, SLE, liver cirrhosis, venous ulcers	Compromised	Death	+
Riazzo et al. (66)	1	2017	M	30	Spain	Soft tissue infection	Deep tissue	Fracture and crush injury	Competent	Recovery	+
Atas et al. (67)	1	2017	F	72	Turkey	Peritonitis	Peritoneal fluid	PD	Compromised	Recovery	+
Matsuura and Sugiyama (68)	1	2018	M	81	Japan	Pneumonia, sepsis	Blood	Pneumonia, stroke, prostate cancer, spinal cord injury	Compromised	Recovery	−
Capretta et al. (69)	1	2018	M	9	USA	Soft tissue infection	Wound	None	Competent	Recovery	−
Botero-García et al. (70)	1	2018	M	69	Columbia	Soft tissue infection	Wound	DM	Competent	Recovery	−
Spiegelhauer et al. (2)	1	2018	F	61	Denmark	Pneumonia, urinary tract infection, diarrhea	Tracheal aspirate, urine, feces	Neurofibromatosis type I, bilateral lung transplant	Compromised	Death	−
Sánchez-Códez et al. (71)	1	2019	M	11	Spain	Catheter-related bacteremia	Blood	Central line, Lennox-Gastaut syndrome, intestinal pseudo-obstruction	Compromised	Recovery	+
Adapa et al. (17)	1	2019	F	48	USA	Peritonitis	Peritoneal fluid	ESRD, DM	Compromised	Recovery	−
Mayfield et al. (72)	1	2019	F	65	USA	Postoperative infection	Wound	Fracture repair	Competent	Recovery	−
Merza et al. (73)	1	2019	F	51	USA	Cholecystitis, septic shock	Bile	None	Competent	Death	−
Broderick et al. (74)	1	2019	M	12	USA	Folliculitis	Skin	None	Competent	Recovery	−
Gupta et al. (75)	1	2019	M	13 m	India	Pneumonia	Blood	None	Competent	Recovery	−
Quan et al. (76)	1	2019	M	55	USA	Soft tissue infection	Tissue	Trauma	Competent	Recovery	+
Keyes et al. (77)	2	2020	M	11	USA	Soft tissue infection	Wound	Penetrating trauma	Competent	Recovery	−
			M	16		Urinary tract infection	Urine	Stage 4 CKD, solitary left kidney, neurogenic bladder	Compromised	Recovery	−
Alosaimi and Kaaki (6)	1	2020	F	50	Saudi Arabia	Catheter-related bacteremia	Blood	ESRD, DM, hemodialysis	Compromised	Recovery	−
Courtois et al. (78)	1	2020	M	8	Argentina	Urinary tract infection	Urine	ALL, chemotherapy	Compromised	Recovery	−
Lonneman et al. (79)	1	2020	F	72	USA	Soft tissue infection	Wound	None	Competent	Recovery	−
Kaushik et al. (80)	1	2020	F	22	USA	Soft tissue infection	Pus, necrotic tissue	None	Competent	Unk	+
Gómez-Arroyo et al. (81)	1	2020	F	72	Spain	Soft tissue infection	Wound	Prosthetic limb	Compromised	Recovery	+
Hassan et al. (16)	1	2020	M	7	India	Peritonitis	Blood	Nephrotic syndrome	Compromised	Recovery	−
Fadeyi et al. (82)	1	2020	M	63	USA	Left hip fracture	Blood	ESRD, dialysis, cirrhosis	Compromised	Death	+
Sng et al. (83)	6	2021	6 M	66	Singapore	Five with bacteremia, one with soft tissue infection	Blood	DM, hypertension, HCC, metastatic lung cancer	Unk	4 recoveries, 2 deaths	Unk
Zayet et al. (18)	6	2021	F	71	France	Peritoneal dialysis peritonitis	Dialysis fluid	Kidney transplantation	Compromised	Recovery	−
			F	19		Corneal abscess with superficial punctate keratitis	Contact lens fluid	None	Competent	Recovery	+
			M	68		Ventilator-associated pneumonia/ARDS	Bronchial aspiration	Mechanical ventilation	Compromised	Death	+
			M	74		Catheter-associated male urinary tract infection	Urine	Urinary catheter, BPH	Compromised	Recovery	-
			M	81		Vascular prosthetic graft infection	Iliofemoral prosthetic vascular graft	Prosthetic graft	Compromised	Recovery	+
			M	84		Catheter-associated male urinary tract infection	Urine	Urinary catheter, BPH	Compromised	Recovery	+
Garza-González et al. (84)	25	2021	16M, 9F	6	Mexico	Bacteremia	Blood	Contaminated TPN formula (outbreak)	Compromised	24 Recoveries, 1 Death	Unk
Malik et al. (85)	1	2021	M	62	Georgia	Endocarditis	Blood	Cardiac disease, pacemaker, hypertension, colostomy	Compromised	Recovery	−
Shaikhain et al. (7)	1	2021	F	74	Saudi Arabia	Pneumonia	Blood	None	Competent	Death	−
Li et al. (19)	1	2021	M	93	USA	Urinary tract infection	Urine	Radiation cystitis, Foley catheter	Competent	Recovery	+
Aarab et al. (86)	1	2021	F	9d	Morocco	Intestinal obstruction	Blood, Cerebrospinal fluid	Hirschsprung disease	Compromised	Recovery	−
Gamon et al. (87)	1	2021	M	55	Germany	Septic shock	Bronchial aspiration	COVID-19 pneumonia	Competent	Recovery	+
King et al. (88)	1	2021	M	21	USA	Osteomyelitis, septic arthritis	Bone, synovial fluid	Trauma	Competent	Recovery	+
Benachinmardi et al. (89)	85	2022	Unk	Unk	India	Unk	Blood, tracheal aspirate, ventriculoperitoneal shunt tip, pus, wound, urine	Hospital outbreak	Unk	85 Recoveries	Unk
Arasu et al. (90)	2	2022	Unk	7	Australia	Septic arthritis	Synovial fluid	Trauma	Competent	Recovery	−
			Unk	3		Septic arthritis	Synovial fluid	Trauma	Competent	Recovery	−
Harper et al. (91)	1	2022	M	34	USA	Sepsis	Blood	Chemotherapy, central venous catheter	Compromised	Unk	−
Anaut et al. (92)	1	2022	Unk	24 wk	Spain	Nosocomial sepsis	Blood	Prematurity	Compromised	Death	−
Householder et al. (93)	1	2022	M	54	USA	Septic arthritis	Synovial fluid	Knee arthroscopy	Competent	Unk	−
Tan et al. (94)	1	2022	M	51	China	Endocarditis	Blood	Cardiac disease	Competent	Recovery	−
Sahu et al. (95)	1	2022	M	10 m	India	Meningoencephalitis	CSF	None	Competent	Recovery	−
Al Shuhoumi et al. (96)	1	2022	F	63	Oman	Cerebral hemorrhage	Rectum	DM, CKD	Competent	Death	+
Dotis et al. (97)	1	2022	M	14.5	Greece	Peritonitis	Peritoneal fluid	Nephrotic syndrome, PD	Competent	Recovery	−
Colangelo et al. (98)	1	2022	F	38	Italy	Catheter-related bacteremia	Blood	DLBCL	Compromised	Recovery	−
Meng et al. (99)	1	2023	F	5 d	China	Unk	Feces	None	Competent	Unk	−
Present case	1	2023	M	60	Saudi Arabia	Back pain and radiculopathy	Spinal disc	None	Competent	Recovery	−

^*a*^ ALL: acute lymphoblastic leukemia; AML: acute myeloid leukemia; CKD: chronic kidney disease; COPD: chronic obstructive pulmonary disease; CSF: cerebrospinal fluid; d: day; DLBCL: diffuse large B-cell lymphoma; DM: diabetes mellitus; ERCP: endoscopic retrograde cholangiopancreatography; ESRD: end-stage renal disease; F: female; HCC: hepatocellular carcinoma; HIV: human immunodeficiency virus; IBD: inflammatory bowel disease; M: male; m: month; MM: multiple myeloma; PD: peritoneal dialysis; PE: pulmonary embolism; RDS: respiratory distress syndrome; RHD: rheumatic heart disease; SIRS, systemic inflammatory response syndrome; SLE: systemic lupus erythematosus; SVI: superficial venous insufficiency; TB: tuberculosis; TPN: total parenteral nutrition; Unk: Unknown; wk: week.

^*b*^ All ages are in years unless otherwise noted.

^*c*^ + indicates the precence of polymicrobial infection, and − indicates the absence of a polymicrobial infection.

The infections occurred across different continents without a discernible pattern: Asia (120 patients), North America (38 patients), Europe (35 patients), South America (31 patients), Australia (2 patients), and Africa (one patient). Of the 227 patients, 88 were males, 48 were females, and 91 were of unreported sex. The patients ranged from 5 days to 93 years of age (median age: 47 years) as follows: eight infants (≤12 months), 15 children (≤12 years), 53 adolescents and adults (12–60 years), 33 older adults (≥60 years), and the rest unknown.

Bacteremia was the main clinical manifestation in 50 patients (22% of all patients), 35 of whom likely acquired the infection from catheter use, with 25 of this subset (catheter-related bacteremia) reported in an outbreak in Mexico caused by the contamination of total parenteral nutrition formula. Furthermore, 28 patients presented with soft tissue or wound infections (12.3% of all patients). Of nine patients who developed peritonitis, seven were on peritoneal dialysis. Furthermore, polymicrobial infection was reported in 31 patients (13.7%), was absent in 73 patients (32%), and was not specified in 123 patients (54%). Regarding outcomes, 196 patients (86%) achieved recovery, whereas 16 patients (7%) died. The outcomes for the remaining 15 patients were unreported.

We identified several key risk factors associated with *L. adecarboxylata* infection. Chronic conditions such as diabetes mellitus and renal failure (end-stage renal disease) were prevalent, and cancer, hepatic cirrhosis, and metabolic syndrome were common. Patients with a history of organ transplant, chemotherapy, or long-term use of medical devices such as central lines and catheters were also at higher risk, whereas a small percentage of patients had no precipitating risk factor to begin with. Overall, these risk factors underscore the complex and multifactorial nature of *L. adecarboxylata* in vulnerable patient populations.

The bacterium exhibited resistance to beta-lactams, specifically ampicillin (as observed in all patients in a report of 85 patients), cephalosporins (e.g., cefazolin, ceftriaxone, and cefuroxime), and aztreonam. It also demonstrated resistance to the aminoglycosides gentamicin and tobramycin, as well as to trimethoprim–sulfamethoxazole (cotrimoxazole), a combination frequently used in treating urinary tract and respiratory infections. The presence of extended-spectrum beta-lactamases (one patient) and carbapenemase (one patient) in some isolates has further exacerbated the difficulty in managing infections, as these enzymes confer resistance to a wide range of beta-lactam antibiotics. Additionally, resistance to amoxicillin–clavulanate, fosfomycin, and piperacillin–tazobactam has been documented.

The main limitation of this literature review was data inconsistency, as different studies reported variable case information that complicated classification and comparison. Additionally, we did not include results on antibiotic sensitivity, as the lack of a standardized antibiotic selection approach and the use of different regimens across patients made comprehensive insights on treatment outcomes difficult to achieve. Furthermore, the decision to continue antibiotics for 6 weeks was based on follow-up imaging and clinical assessment to reassess the need for further treatment. However, the patient was lost to follow-up after the initial treatment, preventing assessment of long-term outcomes or potential complications.

In conclusion, we have presented both a unique case of a patient with spinal *L. adecarboxylata* infection and an extensive related literature review. The microbiological diagnosis of *L. adecarboxylata* presents a challenge as it can be easily misidentified as other species of Enterobacterales. Moreover, although the bacterium has a good susceptibility profile, resistance is emerging. Importantly, large gaps in the literature about the route of infection, risk factors, and best therapeutic regimen remain.
